# Improving Interlaminar Fracture Toughness and Impact Performance of Carbon Fiber/Epoxy Laminated Composite by Using Thermoplastic Fibers

**DOI:** 10.3390/molecules24183367

**Published:** 2019-09-16

**Authors:** Ling Chen, Li-Wei Wu, Qian Jiang, Da Tian, Zhili Zhong, Yan Wang, Hong-Jun Fu

**Affiliations:** 1 Tianjin and Ministry of Education Key Laboratory for Advanced Textile Composite Materials, Tiangong University, Tianjin 300387, China; chenling190605@163.com; 2 School of Textile Science and Engineering, Tiangong University, Tianjin 300387, China; tianda0987@163.com (D.T.); zhongzhili@tjpu.edu.cn (Z.Z.); wymiss9431111@126.com (Y.W.)

**Keywords:** interlaminar fracture toughness, impact performance, thermoplastic polyimide, laminated composite, carbon fiber

## Abstract

The effects of thermoplastic polyimide (PI) and polypropylene (PP) fibers and areal density of toughened layer on interlaminar fracture toughness and impact performance of carbon fiber/epoxy (CF/EP) laminated composites were studied. Mode I interlaminar fracture toughness (G_IC_) was analyzed via double cantilever beam (DCB) tests. When comparing for the toughener type, PI played a positive role in enhancing the mode-I fracture toughness, while PP was not effective due to the less fiber bridge formed during composite curing. The toughening effects of areal density of PI were further investigated by end notched flexure (ENF) testing and low velocity impact testing to better understand the toughening mechanisms. The results revealed that the toughening effect reached its best effectiveness when the areal density of toughened layer was 30 g/m^2^. Compared with the control group, G_IC_ and G_IIC_ of CF/EP laminated composite were increased by 98.49% and 84.07%, and F_max_ and E_e_ were enhanced by 92.38% and 299.08% under low velocity impact. There is no obvious delamination phenomenon on the surface of laminates after low velocity impact, indicating the improved interlaminar and impact performance of laminated composite.

## 1. Introduction

Carbon fiber/epoxy (CF/EP) laminated composites with light weight, good tensile performance, low cost, and ease of operation are widely used in various fields, including the automotive, aerospace, and petrochemical industries [1,2,3,4]. However, poor interlaminar strength is an inherent drawback of any laminated composite due to its low delamination resistance and damage tolerance, particularly under impact loading; this condition significantly reduces the load bearing capacity of laminated composites [5,6,7,8]. Therefore, improving the interlaminar fracture toughness of carbon fiber composite laminates has elicited the attention of researchers.

Thermoplastic materials can be used as a toughener for epoxy resin to improve the mechanical properties, such as tensile strength and modulus, of thermosetting materials [9,10]. The use of thermoplastic materials as a toughener for thermosetting resins has elicited considerable attention. Resin modification using thermoplastic materials can be divided into two major categories: bulk resin modification toughening and inter-/intralaminar toughening [11]. Although resin modification toughening can improve the interlaminar fracture toughness of composites, it results in other issues, such as increased viscosity and the uneven dispersion of the resin [12]. Interlaminar toughening avoids the shortcomings of toughener dispersion in resin modification toughening; thus, it is more suitable for laminated composite production [13].

With regard to inter-/intralaminar toughening, extensive studies have been performed on using yarns with a large fiber diameter to investigate the effect of interlayer toughening [14,15,16]. Yasaee et al., Sun et al. [17,18], and Wang et al. [19] conducted systematic studies on composite laminates interleaved with chopped Kevlar fibers. Their results showed that a crack interface bridged by short Kevlar fibers was the major toughening mechanism. S.N. Yadav [20] investigated how mode II fracture toughness (G_II_) was affected by Kevlar fiber reinforcement in the fracture plane. The results showed that the G_II_ of the investigated composite was enhanced by 1.5 times with short Kevlar reinforcement. In the study of B. del Saz-Orozco et al. [21], polyamide (PA)-interleaved composites exhibited extensive fiber bridging, fiber pullout, and plastic deformation; consequently, the interlaminar fracture toughness value of the studied composites was increased by 59%. V.A. Ramirez [22] studied the modes I and II interlaminar fracture toughness of polyphenylene sulfide nonwoven veils interleaved within unidirectional CF/EP composites. Bond et al. [23] employed the thermosetting adhesive film ahead of the crack path in the mid-plane, which showed an increase in G_IIC_ of 112%, however the resin diffused, and thus good control of its final cured shape was not possible. Lee et al. [24,25] discovered that the interleaving of the non-woven carbon tissue (NWCT) layer significantly increased the Mode II interlaminar fracture toughness, while it did not significantly change the Mode I interlaminar fracture toughness. When the short carbon fiber volume fraction in the NWCT layer was about 10%, the Mode I interlaminar fracture toughness of the NWCT interleaved specimen was 28% higher than that of the CFRP specimen.

On the basis of the aforementioned studies, two thermoplastic fibers, namely, polyimide (PI) and polypropylene (PP), were selected as tougheners in the current study due to their high toughness and strength. The effects of toughener type and the areal density of the toughened layer on fracture toughness and impact properties were discussed. The interlaminar toughness G_IC_ and G_IIC_ of our laminated composite can be increased by 98.49% and 84.07%, respectively, by using the PI fiber nets as a toughening layer. Compared with previous studies, the fabrication efficiency and economic efficiency are both considered, and this toughening method barely influences the original architecture of composite materials. Besides, the proposed toughening method can be a promising solution to the issue of recycling thermoplastic fiber leftovers. This method is a simple and economical means of composite toughening, and it can be used extensively in toughening composite laminates.

## 2. Results and Discussion

### 2.1. Mode I Interlaminar Fracture Toughness of the Laminated Composites

#### 2.1.1. Effect of Toughener Type on the Interlaminar Toughening of the CF/EP Laminated Composites

**Figure 1:**
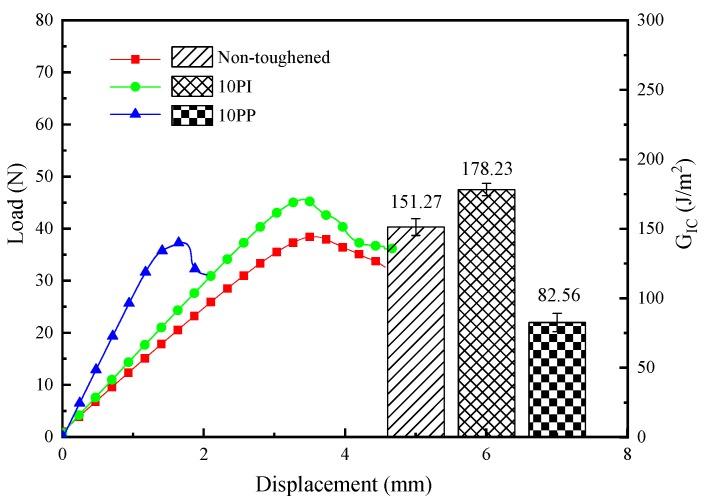
Load–displacement curves and Mode I interlaminar fracture toughness for the non-toughened, 10 PI- and 10 PP- toughened composites.

10 PI- and 10 PP-toughened composites were compared to investigate the effect of toughener types on the interlaminar toughening of the CF/EP laminated composites. The typical load–displacement curves and the calculated G_IC_ are presented in Figure 1. The trend of the load–displacement curves of the three composites follows the same pattern, in which the maximum load (F_max_) of the 10 PI-toughened composite is 45.13 N, i.e., an increase of 24.94% compared with that of the non-toughened composite. The F_max_ of the 10 PP-toughened laminate was 36.22 N, which is similar to that of the non-toughened composite. The maximum displacements (D_max_) of the non-toughened, 10 PI-toughened, and 10 PP-toughened composites are 3.51, 3.34 mm, and 1.92 mm, respectively. Evidently, that of the 10 PP-toughened composite is the smallest. The G_IC_ of the 10 PI-toughened composite is 178.23 J/m^2^, i.e., an increase of 115.88% and 17.82% relative to the PP-toughened and non-toughened composites, respectively. For the aforementioned interlaminar behavior difference, PI and PP exert distinct effects on the interlaminar toughening of the composites. PI fibers play a positive role in enhancing mode I fracture toughness of the CF/EP laminated composites. By contrast, PP fibers are ineffective in increasing interlaminar fracture toughness. The bridging effect of fibers is the primary reason for PI toughening [26,27]. During the loading process, energy is dissipated by debonding between PI fibers and the matrix, deformation and breakage of PI fibers occur, and consequently, the toughness of composites is improved. When PP fibers are used as a toughening layer, they melt into a thin film when the composite is cured at a high temperature. Therefore, fiber bridges are not formed, and the characteristic of fibers is lost. The result is poor interfacial bonding between PP fibers and the resin matrix.

**Figure 2:**
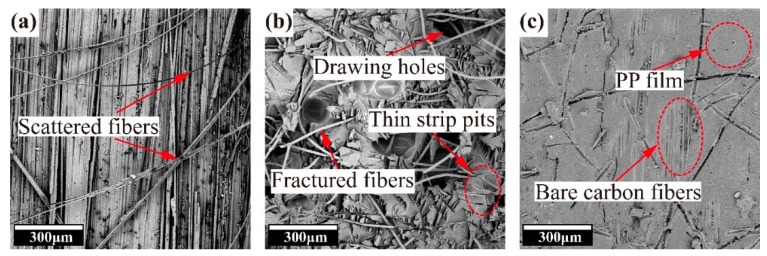
SEM images of delaminated surfaces of composites after DCB testing. (**a**) Non-toughened composite, (**b**) 10 PI-toughened composite, (**c**) 10 PP-toughened composite.

The findings were further confirmed via SEM observations of the delaminated surface after DCB testing (Figure 2). A small amount of broken and scattered carbon fibers were floating on the delaminated surface of the non-toughened composite, as shown in Figure 2a. The fibers at the bottom exhibited a highly ordered arrangement, which indicates that bonding between carbon fibers and the resin is weak. The debonding behavior is the primary reason for the delamination of the non-toughened composite. The delaminated surface of the 10 PI-toughened composite presents a large number of broken PI fibers, and the matrix is filled with drawing holes left by fiber pullout, as shown in Figure 2b. The small strip-shaped pits are the debonding regions between the PI fibers and the resin. A conclusion can be drawn that the 10 PI-toughened layer plays an important role in restraining crack propagation during mode I fracture. The pullout and fracture of PI fibers improve interlaminar toughness by resisting debonding between the fibers and the matrix. In Figure 2c, the PP film is formed after the high-temperature melting of PP fibers. This film exhibits the same continuous phase as epoxy resin. As shown by the exposed ordered carbon fibers, delamination occurs at a weaker interface and decreases the toughening effect [28].

#### 2.1.2. Areal Density Effect of PI on the Interlaminar Toughening of the CF/EP Laminated Composites

**Figure 3:**
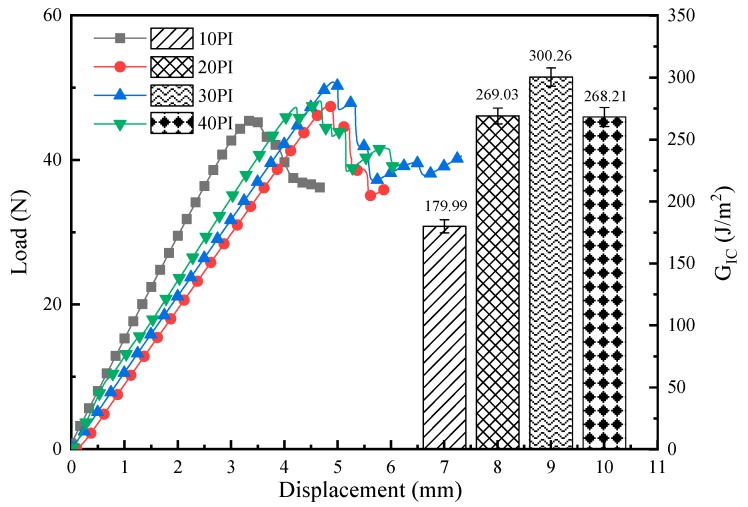
Load–displacement curves and mode I interlaminar fracture toughness for 10 PI-, 20 PI-, 30 PI-, and 40 PI-toughened composites.

To analyze the effect of the areal density of PI fibers, mode I fracture toughness tests were performed on 10 PI, 20 PI, 30 PI, and 40 PI. The results of the load–displacement curves and mode I interlaminar fracture toughness derived from DCB testing are presented in Figure 3. From the load–displacement curves, the F_max_ and D_max_ of the 10 PI-, 20 PI-, and 30 PI-toughened composites increased gradually. However, the F_max_ and D_max_ of the 40 PI-toughened composite decreases by 4.71% and 6.26%, respectively, compared with those of the 30 PI-toughened composite. Similarly, the G_IC_ values of the four composites exhibited the same trend, with an increase in PI areal density. Among the four composites, when the toughening layer is 30 PI, the composite laminates have the largest G_IC_ value compared with other laminates, i.e., an increase of 98.49% relative to the non-toughened composite, which has a G_IC_ of 151.27 J/m^2^. The primary reason for this phenomenon is as follows: as PI areal density increases, fiber bridges tend to be extensively formed and the load-carrying and stress-transferring capacities of the PI network, and consequently, delamination resistance, become stronger. Nevertheless, an excessive areal density of PI fibers leads to the imperfect diffusion of epoxy resin. This phenomenon leaves defects at the interface between the toughened layer and the carbon fiber plies. Consequently, an abnormal decrease in the G_IC_ of the 40 PI-toughened composite indicates that the areal density of the PI fibers reaches the threshold at 30 g/m^2^.

**Figure 4:**
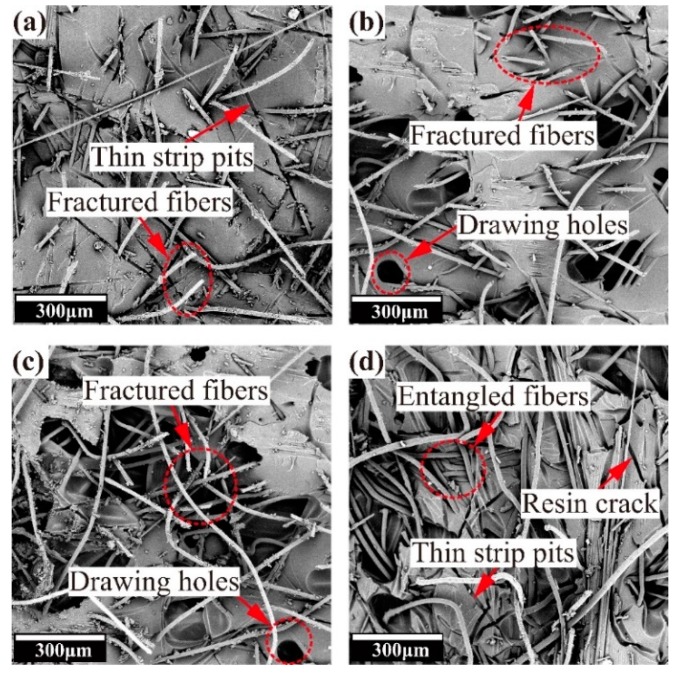
SEM images of delaminated surfaces of (**a**) 10 PI-toughened composite, (**b**) 20 PI-toughened composite, (**c**) 30 PI-toughened composite, and (**d**) 40 PI-toughened composite after DCB tests.

The damage morphologies of the PI-toughened composites with different areal densities after mode I fracture are shown in Figure 4. The comparison of Figure 4a–c shows that more fiber breakage and pullout holes appear on the delaminated surface with an increase in areal density. For the 30 PI-toughened composite, rupture occurs on most fibers and pullout holes expand into large holes. Correspondingly, the energy consumed during crack propagation is the highest, and the effect of crack propagation inhibition is evident. The delaminated surface of the 40 PI-toughened composite presents less broken fibers and holes. Fibers are prone to entangle due to the high content of PI fibers. This condition weakens the bridging effect among fibers and reduces the toughening effect.

### 2.2. Mode II Interlaminar Fracture Toughness of the Laminated Composites

**Figure 5:**
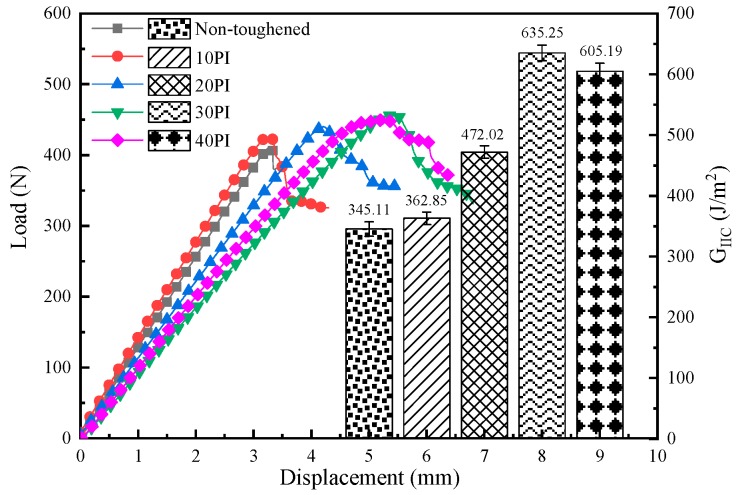
Load–displacement curves and Mode II interlaminar fracture toughness for 10 PI-, 20 PI-, 30 PI-, and 40 PI-toughened composites.

The analysis of mode I interlaminar fracture toughness shows that PI fibers significantly affect G_IC_. Further studies on the areal density effect of PI fibers was conducted via mode II fracture toughness testing of the composites with different PI areal densities as the toughening layer. As shown in Figure 5, when the load of the non-toughened composite reaches F_max_, a fracture occurs and displacement fails to increase; thus, the crack propagates rapidly between layers. However, the fracture behavior of the PI-toughened composites is demonstrated in a completely different manner. With the addition of the PI-toughened layer, displacement continues to increase after the load reaches F_max_. Therefore, the toughness of the composite is improved. With an increase in PI areal density, G_IIC_ initially increases and then decreases, similar to the pattern shown in Figure 3. The G_IIC_ of the 30 PI-toughened composite increases by 84.07% compared with that of the non-toughened composite. The higher the PI areal density, the more PI fibers are bridged between two layers of unidirectional carbon fabrics through the resin. Therefore, higher friction between rough fracture surfaces contributes to an increase in G_IIC_. However, when the areal density of the toughening layer reaches 40 g/m^2^, G_IIC_ evidently decreases because fiber entanglement occurs with high fiber content. This condition affects the contribution of fibers to the toughening effect.

### 2.3. Impact Testing of the Laminated Composites

**Figure 6:**
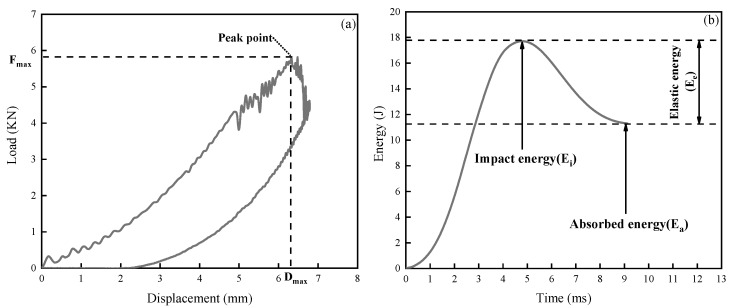
Typical load–displacement (**a**) and energy–time (**b**) curves of toughened laminated composite.

Typical load–displacement and energy–time curves were obtained during impact testing, as shown in Figure 6. With regard to the load–displacement result (Figure 6a), small oscillations occurred during loading, primarily because of matrix cracking and fiber debonding in the toughened layer [29]. After reaching the peak point, the evident fluctuations were due to damage propagation in the composites, and fiber failure occurs during this stage. The load that corresponds to the peak point is the maximum load (F_max_) and the displacement is the maximum displacement (D_max_). In the energy–time curves (Figure 6b), absorbed energy (E_a_) is derived from the value of the horizontal part of the energy curve until the energy peak. Impact energy (E_i_) corresponds to the energy peak. Elastic energy (E_e_) is defined by the difference between impact energy and absorbed energy [30].

**Figure 7:**
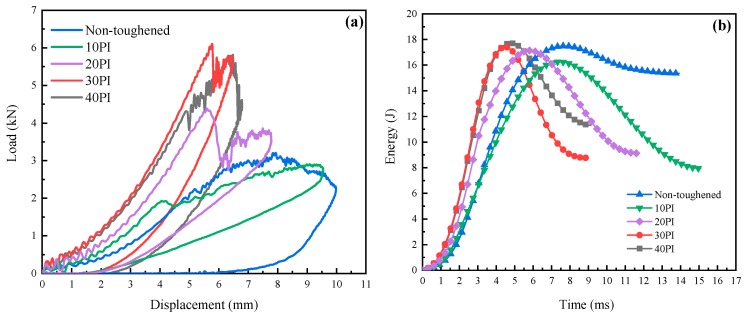
Effect of areal densities of PI interleave on the impact properties of composite laminates: (**a**) load–displacement curves, (**b**) energy–time curves.

The average value of E_a_, E_e_, F_max_, and D_max_ of six specimens for each type of laminated composites during impact tests are summarized in Table 1. The load–displacement and energy–time curves of the non-toughened and toughened composite laminates are presented in Figure 7. The trends of the load–displacement curves of the five composites in Figure 7a are similar, and all the curves are closed; such results imply that penetration does not occur [31]. The variation of F_max_ and D_max_ can be clearly seen from the curve. Compared with that of the non-toughened laminates, the F_max_ change of 10 PI is negligible because of the slight bridging effect and low bonding to resins when the areal density of the toughened layer is low. Delamination occurs after the rapid propagation of interlaminar cracks; thus, D_max_ is increased. The F_max_ of 30 PI exhibits the greatest enhancement, with an increase of 92.38% compared with that of the non-toughened composite. Meanwhile, the F_max_ of the 40 PI laminate presents a downward trend. Under impact loading, the 30 PI-toughened layer significantly improves the interface between the matrix and the carbon fibers. Instead of being damaged, the composite is well-compressed and densified. Therefore, the resistance of delamination propagation is improved and D_max_ decreases. Higher loads are transferred to the carbon fibers via the interface, and mechanical strength is improved. However, when the areal density of the toughened PI fibers increases to 40 g/m^2^, binding and entanglement occur between fibers, and toughening properties are decreased. As shown in Figure 7b, the E_a_ of the toughened composites is lower than that of the non-toughened laminates because most of the impact energy of the toughened laminates is consumed via impactor rebound [29]. Table 1 shows that the 30 PI-toughened composite has the highest E_e_, i.e., 299.08% higher than that of the non-toughened laminates. This result indicates the improvement of interlaminar toughness.

### 2.4. Damage Analysis

**Figure 8:**
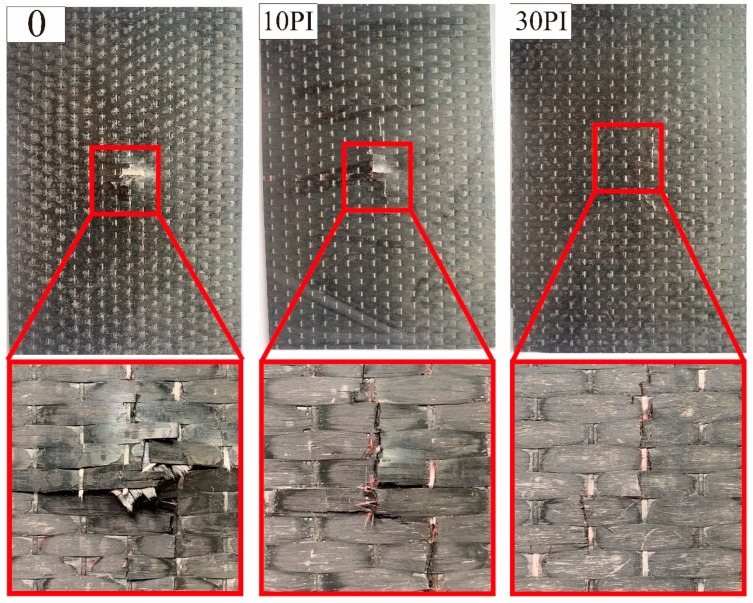
Damage morphologies of non-toughened, 10 PI-toughened, and 30 PI-toughened composites from back view.

The damage morphologies of the non-toughened, 10 PI-toughened, and 30 PI-toughened composites after impact loading are shown in Figure 8. The non-toughened composite presents severe delamination areas and fiber breakage. Fiber breakage is the primary damage form of the 10 PI-toughened composite with less evident delamination. The 30 PI-toughened composite achieves the highest structural integrity at the impact point, and only a few signs of carbon fiber damage are observed. This finding proves that this composite can bear the majority of the load induced by impact. The use of PI fibers as a toughener can considerably reduce the damage evolution of laminated composites. The toughened layer can improve delamination resistance by cutting off crack paths and establishing bridges through crack regions [32]. These results are consistent with those presented in the previous sections.

## 3. Experiments

### 3.1. Materials

Carbon fiber unidirectional composite laminates with different toughening layers were prepared. For all the specimens, the resin used was bisphenol A epoxy resin E-51(128) from Guangdong Suixin Chemical Co., Ltd. The curing agent was EMI-2,4, which was supplied by Tianjin Dahua Technology Co., Ltd. (Tianjin, China) Methyl tetrahydrophthalic anhydride promoter was provided by Taizhou Jiangping Petrochemical Co., Ltd. (Taizhou, Zhejiang, China). Unidirectional T300 carbon fiber fabric was produced by Toray (Central District, Tokyo, Japan), with an areal density of 350 g/m^2^. Thermoplastic PI fiber with high strength, high modulus, and high toughness was provided by Jiangsu Aoshen New Materials Co., Ltd. (Jiangsu, Rudong, China). PP fibers with a low melting point and high toughness were provided by Zibo Lone Fiber Company (Zibo, Shandong, China). PI and PP were cut into 10~15 mm staples and processed with a carding machine to form PI and PP nets with randomly arranged fibers. Different areal densities were prepared as different weights of toughened layer per square meter.

### 3.2. Composite Preparation

**Figure 9:**
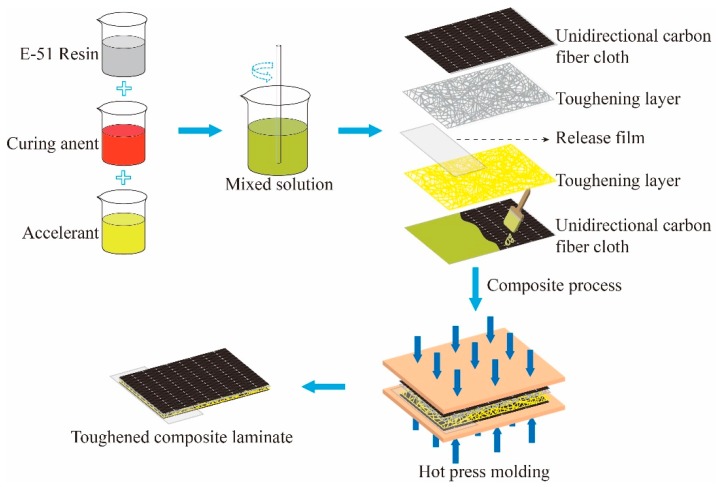
Schematic view of manufacturing toughened composite laminates.

**Figure 10:**
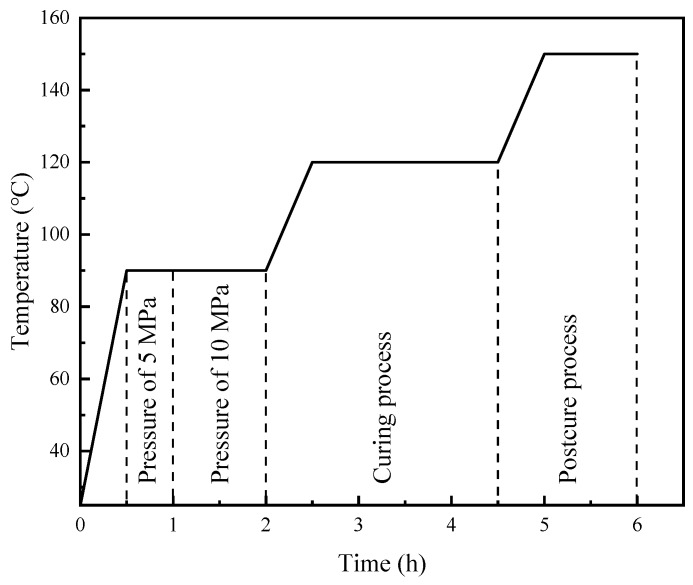
Curing program of laminated composite under hot pressing.

The CF/EP laminated composites were manufactured via hand lay-up, as illustrated in Figure 9, by stacking two layers of unidirectional T300 carbon fiber fabric and a certain number of layers of PI and PP nets. PI and PP nets were made by combing the PI and PP staples into a loose assembly by the carding machine, and then hot pressed under pressure of 10~15 Pa to make them denser. The morphology of toughening layer is illustrated in Figure 9 in the form of randomly arranged fiber assembly. A sheet of release film with 20 µm thickness was inserted between the layers to serve as the initial pre-crack for modes I and II delamination toughness testing. Epoxy resin, curing agent, and accelerant were mixed at a weight ratio of 100:70:1 and evacuated for 20 min at 80 °C to remove bubbles and reduce the viscosity of the resin. In this manner, full impregnation of the unidirectional carbon fiber fabric was ensured. After resin coating was finished, the CF/EP laminates were placed on a plate vulcanizer for hot pressing. The curing program is illustrated in Figure 10. When temperature reached 90 °C, a pressure of 5 MPa was applied and maintained for 0.5 h to ensure the diffusion and infiltration of the resin. The resin was fully infiltrated by applying a pressure of 10 MPa for 1 h, followed by heating to 120 °C for 2 h, and maintaining at 150 °C for 1 h to complete the curing stage. PI nets were carded into four specifications with areal densities of 10, 20, 30, and 40 g/m^2^, labeled as 10 PI, 20 PI, 30 PI, and 40 PI, respectively. PP nets were carded with an areal density of 10 g/m^2^, referred to as 10 PP. CF/EP laminated composites without a toughener were also fabricated as control samples.

### 3.3. Mode I Interlaminar Test

**Figure 11:**
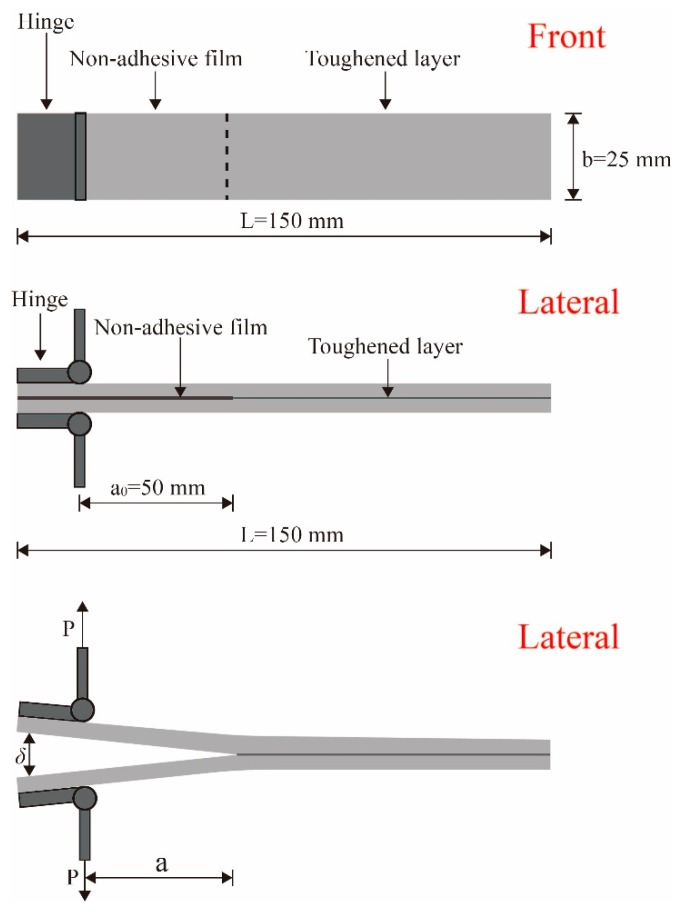
Dimensions and schematics of laminated composite and double cantilever beam (DCB) testing (dimensions are in mm).

Mode I interlaminar fracture toughness (G_IC_) was measured following ASTM D5528 [33]. A double cantilever beam (DCB) test was performed using a universal testing machine (UTM; Instron 5965) with 200 N capacity load cell. Ten specimens from each type of laminated composites were tested and average values were calculated for comparison. The size of the samples was 150 × 25 × 3 mm^3^, and the initial crack length was 50 mm (a_0_), as shown in Figure 11. Load was applied at a constant rate of 2.0 mm/min, and the load–displacement curve was recorded during the test. In accordance with ASTM standard, G_IC_ can be calculated as

(1) $$G_{IC}=3P\delta /2b\left(a+\left|\Delta \right|\right)$$

where *P* is the applied load, *δ* is the load point displacement, *b* is the width of a specimen, *a* is the delamination length, and Δ is the corrective factor for crack length [34].

### 3.4. Mode II Test

**Figure 12:**
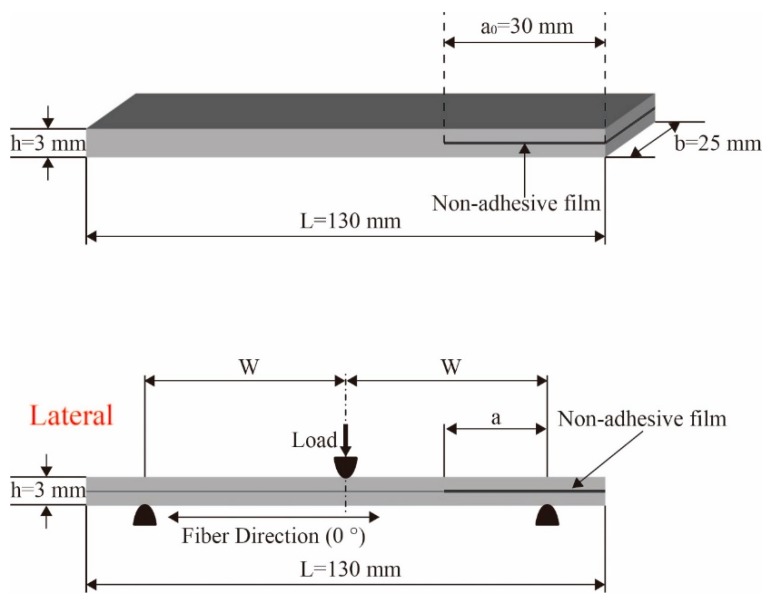
Dimensions and schematic of laminate specimen and the end notched flexure (ENF) test (dimensions are in mm).

Mode II interlaminar fracture toughness (G_IIC_) was obtained by performing end notched flexure (ENF) test in accordance with the test standard ASTM D-3878 [35]. The ENF test was conducted using a UTM (Instron 5965) with 1 kN capacity load cell, the size of the ENF samples was 130 × 25 × 3 mm^3^, and the initial crack length was 30 mm (a_0_), as shown in Figure 12. Ten specimens from each type of laminated composites were tested and average values were calculated for comparison. Load was applied at a constant rate of 0.3 mm/min. In accordance with ASTM D-3878, G_IIC_ can be calculated as

(2) $$G_{IIC}=9a^{2}P\delta /2b\left(2W^{3}+3a^{3}\right)$$

where *P* is the applied load, *δ* is the load point displacement, *b* is the width of a specimen, *a* is the delamination length, and where W is the half-span length.

### 3.5. Impact Test

**Figure 13:**
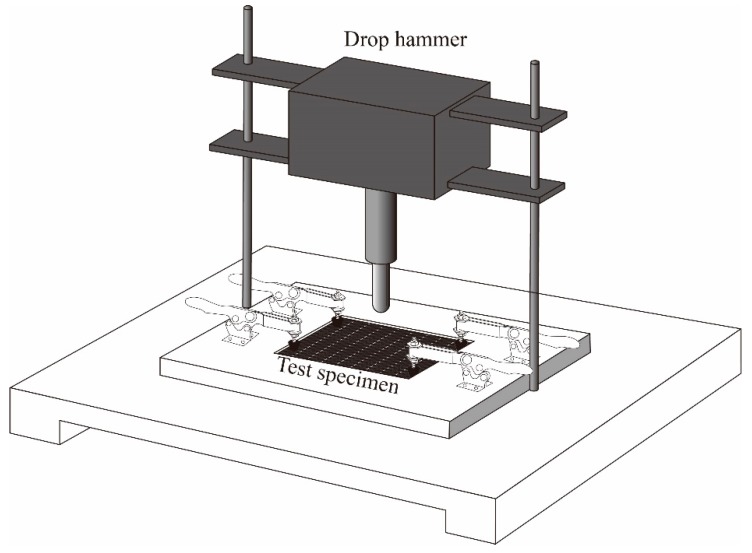
Schematic of low velocity impact test.

Impact and post-impact compressive tests were conducted in accordance with the standard ASTM D7136 [36]. Drop hammer impact test was performed on an Instron Dynatup 9250 HV testing machine. The diameter and weight of the hemispherical hammer were 12.7 mm and 6.5 kg, respectively. The ratio of impact energy to sample thickness was 6.7 J/mm. The impact energy was changed by adjusting the height of the drop hammer. The effective testing area was 125 × 75 mm^2^ (Figure 13). The test machine was equipped with an anti-secondary impact device to prevent the hammer head from repeatedly impacting the specimen. Six specimens from each type of laminated composites were tested and average values were calculated for comparison.

### 3.6. Morphology

Damage morphology was observed after DCB testing via scanning electron microscopy (SEM) (Phenom Pure, Thermo Fisher Scientific, Waltham, MA, USA). Samples that measured approximately 5 × 5 mm^2^ were cut from the specimens after DCB testing at 30 mm from the pre-crack position and were processed via gold sputtering to avoid electrostatic charging.

## 4. Conclusions

In this study, two types of thermoplastic fibers (PI and PP) were added to CF/EP laminated composites as tougheners, and the effects of toughener type and the areal density of the toughening layer were investigated. The conclusions are as follows:

1. Comparing the effects of two types of tougheners, the addition of PI fiber significantly increased the interlaminar toughness, while the addition of PP fiber reduced the interlaminar toughness. Interlaminar cracking was hindered by the debonding between PI fibers and matrix, the deformation and fracture of PI fibers, which greatly improves the interlaminar toughness. By contrast, PP fibers were molten into a continuous phase without the formation of fiber bridge and the interface between melted PP fibers and resin matrix was poor bonding, which reduces the interlaminar toughness.
2. In modes I and II of interlaminar fracture toughness testing, G_IC_ and G_IIC_ first increased and then decreased with the increase of the areal density of the PI-toughened layer. G_IC_ and G_IIC_ of composite laminates reached their maximum values at 30 PI, which increased by 98.49% and 84.07%, respectively, compared with those of the non-toughened composite laminates. However, when areal density reached 40 g/m^2^, G_IC_ and G_IIC_ presented a downward trend due to the entanglement of fibers and the insufficient diffusion of epoxy resin in the toughened layer.
3. In low-velocity impact testing, when the toughened layer is 30 PI, F_max_ and E_e_ increase by 92.38% and 299.08%, respectively, compared with the non-toughened composite laminates. Moreover, the damage morphology after low-velocity impact testing showed that severe delamination areas and fiber breakage did not occur in the 30 PI-toughened composite, and only a few signs of carbon fiber damage were observed, indicating that the 30 PI toughened layer had the best toughening effect.

## Figures and Tables

**Table 1:** Parameters obtained from low velocity impact tests.

Sample	F_max_ (KN)	D_max_ (mm)	E_a_ (J)	E_e_ (J)
Non-toughened	3.15	7.90	15.34	2.18
10PI-toughened	2.88	9.38	7.96	8.35
20PI-toughened	4.34	5.60	9.15	8.03
30PI-toughened	6.06	5.77	8.78	8.70
40PI-toughened	5.80	6.49	11.30	6.45
